# Synthesis, Characterisation and Reactions of Phosphine-Substituted Alkynylboronates and Alkynyltrifluoroborate Salts

**DOI:** 10.3390/molecules191221324

**Published:** 2014-12-18

**Authors:** Jérôme F. Vivat, Sylvestre P. J. T. Bachollet, Harry Adams, Joseph P. A. Harrity

**Affiliations:** Department of Chemistry, University of Sheffield, Sheffield S3 7HF, UK; E-Mails: vivat.jerome@gmail.com (J.F.V.); s.bachollet@sheffield.ac.uk (S.P.J.T.B.); h.adams@shef.ac.uk (H.A.)

**Keywords:** alkynylboronate, trifluoroborate salt, conjugation, phosphine, cross-coupling

## Abstract

The synthesis and structural characterisation of phosphine-substituted alkynylboronates is reported. A P(III)-centred alkynylboronate (**2**) was prepared that showed little evidence for the conjugation of the P-lone pair to the boron via the alkyne π-system, as judged by X-ray crystallography studies of **2** and a related P(V) compound, **3**. In addition, corresponding alkynyltrifluoroborate salts were prepared that showed improved stability by comparison to their boronic ester counterparts. These salts undergo Pd-catalysed cross-coupling reactions with aryl halides.

## 1. Introduction

Since their discovery by Matteson in 1960 [1], alkynylboronates have proven to be a versatile and popular class of synthetic intermediates in organic synthesis. They combine the traditional nucleophilic properties of alkyne-based organometallics and, as such, undergo metal catalysed cross-coupling [2,3,4], as well as addition to aldehydes and imines [5]. Moreover, these reagents are subject to chemistry at the triple bond. For example, hydrogenation [6], metallation [7,8], metathesis [9] and various cycloaddition reactions [10,11,12,13,14,15,16,17,18,19,20] have all been reported.

**Figure 1:**
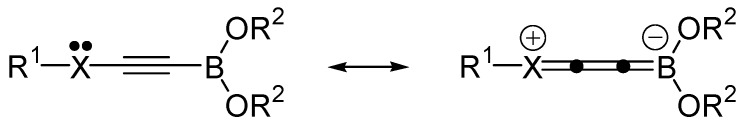
Heteroatom-substituted alkynylboronates.

Whilst a range of reactions of alkynylboronates are now documented, there is relatively little in the literature regarding heteroatom-substituted alkynylboronates of the type shown in Figure 1. Indeed, an early example of an ynol ether-derived alkynylboronate [21] suggested that these compounds were unstable, and this precluded extensive studies into their synthetic chemistry and their structural characterisation. We wanted to explore the chemistry of this type of alkyne and, in particular, the potential for the conjugation of the heteroatom to the boron centre (Figure 1). Evidence for related mesomeric B-C π-bonding in alkynylboronates has been documented by Yamamoto [22], and it seemed feasible that this would be enhanced by the inclusion of an appropriate Lewis basic donor. We report herein our studies on the synthesis, characterisation and reactions of phosphine-substituted alkynylboronates and their closely-related trifluoroborate salts.

## 2. Results and Discussion

**Scheme 1:**
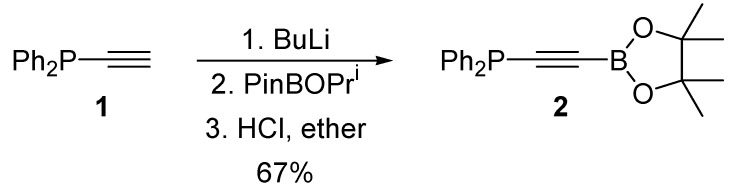
Synthesis of a phosphine substituted alkynylboronate.

**Figure 2:**
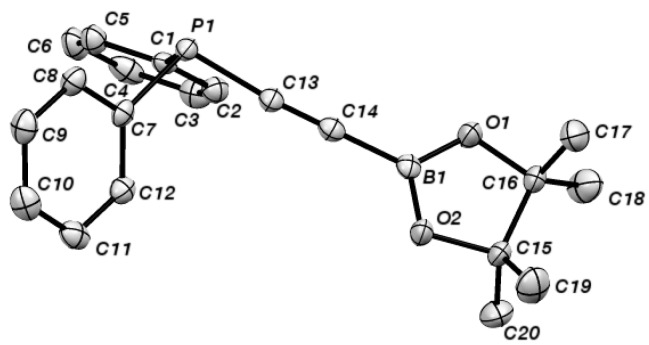
ORTEP diagram of alkyne **2**. H-atoms have been omitted for clarity.

The most convenient method of generating alkynylboronates is via the appropriate alkynyllithium reagent, following the procedure of Brown [23]. Indeed, we were pleased to find that ethynyldiphenylphosphine 1 could be smoothly transformed into the desired alkynylboronate (**2**) in good overall yield when this approach was employed (Scheme 1). Alkyne **2** was found to be highly sensitive to protodeboronation induced by exposure to air and moisture; however, we were able to grow crystals of **2** suitable for single-crystal X-ray diffraction analysis; the structure is shown in Figure 2, and selected bond lengths and bond angles are given in Table 1.

**Table 1:** Selected bond lengths (Ǻ) and angles (°) for **2**.

2	
C(13A)-C(14A)	1.203(4)
B(1A)-C(14A)	1.531(4)
P(1A)-C(13A)	1.761(3)
B(1A)-C(14A)-C(13A)	177.8(3)
P(1A)-C(13A)-C(14A)	175.5(2)
O(1A)-B(1A)-O(2A)	114.8(2)
O(1A)-B(1A)-C(14A)	122.0(2)
O(2A)-B(1A)-C(14A)	123.2(2)
C(7A)-P(1A)-C(1A)	102.19(12)
C(1A)-P(1A)-C(13A)	100.56(13)
C(7A)-P(1A)-C(13A)	100.46(12)

The X-ray crystal structure showed that **2** exhibits typical C-C and C-B bond lengths for an alkynylboronate [24]. Moreover, the C-P bond length is marginally longer than those found in related alkynylphophonium salts, where conjugation is not possible [25]. Furthermore, inspection of the selected bond angles shows that the alkyne is close to linear and that the boron atom is contained within a trigonal structure (sum of internal bond angles: ~360°). In contrast, the sum of the internal bond angles around phosphorus is ~303°, and it therefore adopts a trigonal pyramidal geometry. Taking this data into account, while the P-C-C-B array in **2** is linear and the structure adopts a conformation that provides the potential for conjugation, it appears that delocalisation of the P-lone pair is only weak in this system. These observations mirror those of Marder and co-workers in their studies of alkynylboranes [26].

**Scheme 2:**
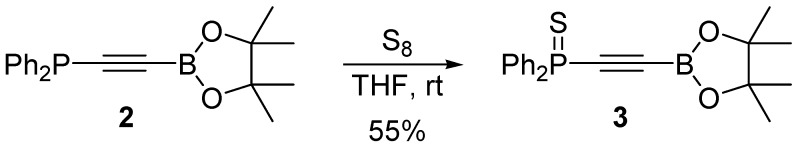
Synthesis of phosphine sulphide.

**Figure 3:**
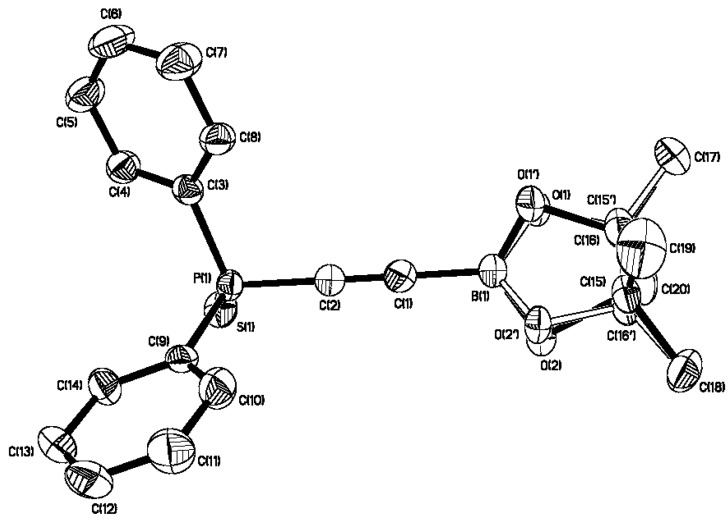
ORTEP diagram of alkyne **3**. H-atoms have been omitted for clarity. O1-O2-C15-C16 were found to be disordered and refined to an occupancy of 49%–51%.

We next decided to explore some reactions at the P-centre of **2** and attempted to generate the corresponding phosphine oxide by oxidation. Unfortunately, this led to rapid decomposition, and only ethynyldiphenylphosphine oxide could be recovered. In contrast, treatment of **2** with elemental sulphur in anhydrous THF provided the corresponding phosphine sulphide **3** in an acceptable yield (Scheme 2). Alkyne **3** was also found to be sensitive to protodeboronation, once again; however, we were able to grow crystals of **3**, and the X-ray structure is shown in Figure 3; selected bond lengths and bond angles are given in Table 2.

**Table 2:** Selected bond lengths (Ǻ) and angles (°) for **3**.

3	
C(1)-C(2)	1.197(4)
B(1)-C(1)	1.544(5)
P(1)-C(2)	1.770(3)
B(1)-C(1)-C(2)	179.3(4)
P(1)-C(2)-C(1)	176.7(3)
O(1)-B(1)-O(2)	114.7(4)
O(1)-B(1)-C(1)	122.1(4)
O(2)-B(1)-C(1)	123.0(4)
C(9)-P(1)-C(2)	103.82(16)
C(3)-P(1)-C(2)	104.20(16)
C(3)-P(1)-C(9)	106.05(14)

The X-ray crystal structure shows that **3** exhibits similar C-C, C-B and C_alkyne_-P bond lengths to those measured in **2**, and again, the alkyne is relatively linear. The boron atom remains within a trigonal structure (sum of internal bond angles: ~360°), whilst the sum of the internal bond angles of the three hydrocarbon groups around phosphorus has expanded to ~314°, and the structure adopts a distorted tetrahedral geometry. This data shows that the oxidation of P(III) in **2** has little effect on the bond lengths and angles in the alkynylboronate motif, providing further evidence for minimal conjugation of the P-lone pair with the boronate moiety in **2**.

**Scheme 3:**
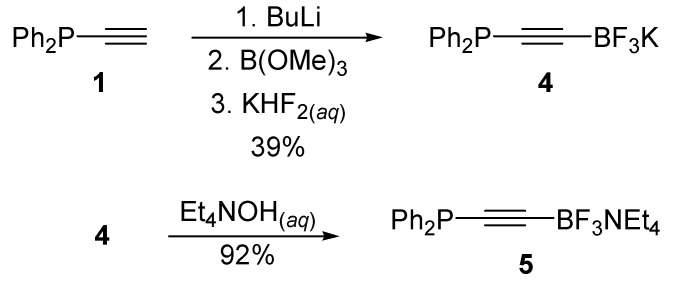
Alkynyltrifluoroborate synthesis.

The air and moisture sensitivity of compounds **2** and **3** prompted us to examine the chemistry of the analogous trifluoroborate salts. Aromatic trifluoroborates have recently emerged as practical alternatives to boronic acid derivatives for cross-coupling and functionalisation reactions [27,28]. These compounds are typically air and moisture stable crystalline solids and usually generated from the corresponding boronic acid derivatives after treatment by KHF_2_. In this event, we were able to transform ethynyldiphenylphosphine **1** to the corresponding potassium tetrafluoroborate **4**, albeit in modest yield. Moreover, we were able to efficiently generate the more soluble tetraethylammonium derivative **5** by counterion exchange (Scheme 3).

**Scheme 4:**
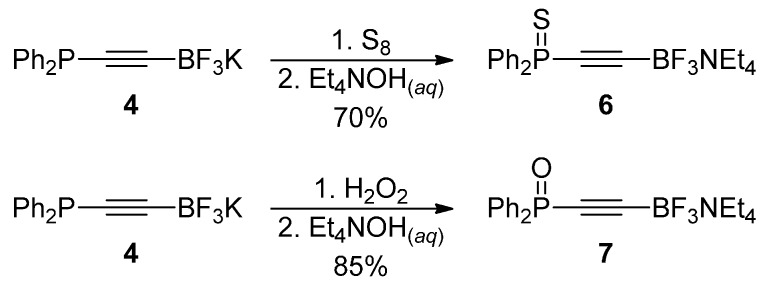
Alkynyltrifluoroborate oxidation chemistry.

We were pleased to find that compounds **4** and **5** showed improved stabilities over boronic ester analogue **2** and set out to investigate the oxidation chemistry of the P-centre in this molecule. Indeed, we found that we were able to prepare both phosphine sulphide **6** and phosphine oxide **7** from this compound in good yields (Scheme 4).

**Scheme 5:**
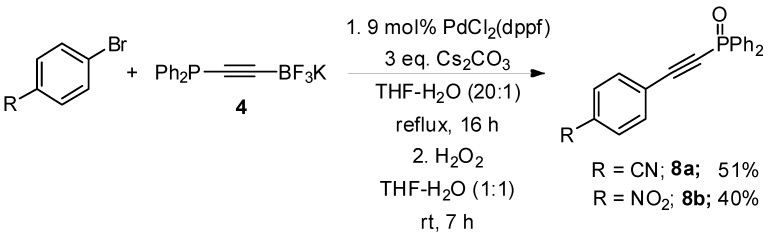
Alkynyltrifluoroborate cross-coupling chemistry.

Our final objective was to confirm the potential of these substrates for further organic synthesis, and we chose to investigate the chemistry of trifluoroborate salt **4**. Preliminary studies towards Lewis acid promoted and directed cycloadditions of 2-pyrones and 1,2,4-triazines [29,30,31] were disappointing, returning protodeborylated alkyne in all cases attempted. However, we were more successful in performing cross-coupling reactions. As shown in Scheme 5, Pd-catalysed cross-coupling with electron-deficient aryl bromides proceeded in a modest yield to provide the corresponding phosphine oxide after work-up with hydrogen peroxide [32,33].

## 3. Experimental Section

### 3.1. General Remarks

All reactions were conducted in an oven or flame-dried glassware under an inert atmosphere of dry nitrogen. Flash chromatography was performed on silica gel (Fluorochem Davisil silica gel 43–60). The solvent system used was a gradient of petroleum ether (40–60), increasing in polarity to ethyl acetate. Thin layer chromatography (TLC) was performed on aluminium-backed plates pre-coated with silica (0.2 mm, Merck DC-alufolien Kieselgel 60 F_254_), which were developed using standard visualizing agents: ultraviolet light or potassium permanganate. 

^1^H-NMR spectra were recorded on a Bruker AV-250 (250 MHz) and AV-400 (400 MHz). Chemical shifts are reported in ppm with the solvent resonance as the internal standard (CHCl_3_: δ 7.26 ppm). ^13^C-NMR spectra were recorded on a Bruker AC-250 (62.9 MHz) and AMX-400 (100.5 MHz) with complete proton decoupling. Chemical shifts are reported in ppm with the solvent resonance as the internal standard (CDCl_3_: δ 77.0 ppm).

Infrared (FTIR) spectra were recorded on a Perkin Elmer Paragon 100 FTIR spectrophotometer, ν_max_ in cm^−1^. Bands are characterized as broad (br), strong (s), medium (m) and weak (w). Samples were recorded as thin films using sodium chloride plates. 

Low resolution mass spectra were recorded on Micromass Autospec, operating in E.I., C.I. or FAB mode; or a Perkin-Elmer Turbomass Benchtop GC-MS operating in either E.I. or C.I mode. High-resolution mass spectroscopy (HRMS), recorded for accurate mass analysis, were performed on either a MicroMass LCT operating in Electrospray mode (TOF ES^+^) or a MicroMass Prospec operating in either FAB (FAB^+^), EI (EI^+^) or CI (CI^+^) mode.

Melting points were recorded on a Gallenkamp melting point apparatus and are uncorrected.

### 3.2. Synthesis of Diphenyl((4,4,5,5-tetramethyl-1,3,2-dioxaborolan-2-yl)ethynyl)phosphine **2**

To a solution of ethynyldiphenylphosphine [34] **1** (1.00 g, 4.76 mmol) in diethyl ether (20 mL) at 0 °C was added a 2 M solution of *n*-butyllithium in hexane (2.38 mL, 4.76 mmol). The reaction was stirred at 0 °C for 30 min before 2-isopropoxy-4,4,5,5-tetramethyl-1,3,2-dioxaborolane (0.97 mL, 4.76 mmol) was added. The reaction was then stirred for 2 h before an anhydrous solution of hydrogen chloride in diethyl ether (1.3 eq, 1 M) was added and the reaction stirred for a further 30 min. The salts were removed by filtration under nitrogen and washed with diethyl ether. The volatiles were removed *in vacuo*. Diethyl ether was added to the residue, and crystallisation was induced by slow evaporation of the solvent using a nitrogen flow (1.07 g, 67%). M.p. 54–56 °C. (dec.). ^1^H-NMR (250 MHz, CDCl_3_): δ 1.31 (12H, s, CH_3_), 7.33–7.38 (6H, m, CH), 7.57–7.66 (4H, m, CH). ^13^C-NMR (62.9 MHz, CDCl_3_): δ 24.7, 47.4, 84.6, 128.6 (d, *J* = 8.0 Hz), 129.1, 133.0 (d, *J* = 21.0 Hz), 135.0. ^31^P-NMR (101 MHz, CDCl_3_): δ −32.5. FTIR: 3055 (m), 2979 (s), 2930 (m), 2139 (s), 1436 (s), 1324 (s) cm^−1^. HRMS calcd. for C_20_H_22_BO_2_P: 336.1450. Found: 336.1465.

### 3.3. Synthesis of Diphenyl((4,4,5,5-tetramethyl-1,3,2-dioxaborolan-2-yl)ethynyl)thiophosphine **3**

Diphenyl((4,4,5,5-tetramethyl-1,3,2-dioxaborolan-2-yl)ethynyl)phosphine **2** (500 mg, 1.48 mmol) was dissolved in THF, and sulphur (142 mg, 4.44 mmol) was added. The reaction was stirred at room temperature for 48 h. THF was removed by cannula filtration. After evaporation of the solvent, the residue was dissolved in anhydrous diethyl ether and the product crystallised by slow evaporation of the solvent using a nitrogen flow (300 mg, 55%). M.p. 75–77 °C (dec.). ^1^H-NMR (250 MHz, CDCl_3_): δ 1.30 (12H, s, CH_3_), 7.39–7.51 (6H, m, CH), 7.88–7.98 (4H, m, CH). ^13^C-NMR (62.9 MHz, CDCl_3_) δ 24.6, 85.3, 94.5, 128.6 (d, *J* = 15.0 Hz), 131.0 (d, *J* = 11.0 Hz), 132.0, 133.7. ^31^P-NMR (101 MHz, CDCl_3_): δ 20.3. FTIR: 3055 (w), 2979 (m), 2925 (m), 2055 (m), 1438 (s), 1348 (s), 1308 (s) cm^−1^. HRMS calcd. for C_20_H_22_BO_2_PS: 368.1171. Found: 368.1187. 

### 3.4. Synthesis of Potassium ((Diphenylphosphino)ethynyl)trifluoroborate **4**

To a solution of ethynyldiphenylphosphine **1** (1.0 g, 4.76 mmol) in THF (20 mL) at 0 °C was added a 2.2 M solution of *n*-butyllithium in hexane (2.16 mL, 4.76 mmol). After 30 min, trimethyl borate (0.8 mL, 7.14 mmol) was added and the reaction mixture stirred at this temperature for 1 h, then a saturated aqueous solution of KHF_2_ (2.23 g, 28.56 mmol) was added at −10 °C. After 1 h, the reaction was allowed to warm to room temperature and stirred for a further hour. The organic phase was separated, dried over MgSO_4_ and concentrated *in vacuo*. The residue was purified by flash chromatography on silica gel (CH_3_CN/DCM: 1/1) to give potassium ((diphenylphosphino)ethynyl)-trifluoroborate (0.589 g, 39%) as a colourless solid. M.p. 80–82 °C (dec.). ^1^H-NMR (250 MHz, d^6^-DMSO): δ 7.28–7.40 (6H, m, Ar-H), 7.53–7.64 (4H, m, Ar-H). ^13^C-NMR (62.9 MHz, d^6^-DMSO): δ 128.8, 129.0, 129.1, 132.1, 132.4. ^31^P-NMR (101.1 MHz, d^6^-DMSO) δ −33.2. ^19^F-NMR (376.5 MHz, CDCl_3_) δ −136.5. FTIR: 3056 (w), 1478 (m), 1434 (m), 986 (s) cm^−1^. HRMS calcd. for C_14_H_10_BF_3_K_2_ [Ph_2_CCBF_3_K_2_^+^]: 354.9845 Found: 354.9839.

### 3.5. Synthesis of Tetraethylammonium ((Diphenylphosphino)-ethynyl)trifluoroborate **5**

A 25% by volume solution of tetraethylammonium hydroxide in water (0.3 mL, 0.53 mmol) was added to a suspension of potassium ((diphenylphosphino)-ethynyl)trifluoroborate **4** (155 mg, 0.49 mmol) in a 4:1 mixture of DCM and water (2.5 mL). The biphasic mixture was stirred for 30 min, and by this time, all of the starting material had dissolved. The organic layer was separated and the aqueous layer washed with DCM (2 × 5 mL). The combined organic fractions were dried over MgSO_4_ and the solvent evaporated to give tetraethylammonium ((diphenylphosphino)-ethynyl)trifluoroborate (183 mg, 92%) as a colourless solid. M.p. 59–68 °C (dec.). ^1^H-NMR (250 MHz, CDCl_3_): δ 1.14 (12H, app. tt, *J* = 7.0 Hz, *J* = 2.0 Hz, CH_3_), 3.09 (8H, q, *J* = 7.0 Hz, CH_2_), 7.24–7.34 (6H, m, Ar-H), 7.59–7.70 (4H, m, Ar-H). ^13^C-NMR (62.9 MHz, CDCl_3_): δ 7.3, 52.4, 128.3–128.6 (m, 2C), 130.8 (d, *J* = 11.5 Hz), 132.5 (d, *J* = 20.0 Hz), 137.6 (d, *J* = 6.5 Hz). ^31^P-NMR (101.1 MHz, CDCl_3_) δ −33.8. FTIR: 2991 (w), 1480 (m), 1440 (m), 1014 (w), 1484 (m) cm^−1^. HRMS calcd. for C_14_H_10_BF_3_P [Ph_2_PCCBF_3_^−^]: 277.0557. Found: 277.0565.

### 3.6. Synthesis of Tetraethylammonium ((Diphenylphosphorothioyl)-ethynyl)trifluoroborate **6**

Sulphur (85 mg, 2.66 mmol) was added to a suspension of potassium ((diphenylphosphino)ethynyl)trifluoroborate **4** in THF (1 mL), and the mixture was stirred at room temperature for 24 h. The solution was filtered via a filter cannula and the solvent evaporated. The residue was then suspended in a 4:1 mixture of DCM and water (3 mL), and a 25% by volume solution of tetraethylammonium hydroxide in water (0.36 mL, 0.73 mmol) was added to the reaction mixture. The biphasic mixture was stirred for 30 min. The organic layer was separated and the aqueous layer washed with DCM (2 × 5 mL). The combined organic fractions were dried over MgSO_4_ and the solvent evaporated to give tetraethylammonium ((diphenylphosphorothioyl)ethynyl)-trifluoroborate (120 mg, 70%) as a yellow solid. M.p. 97–99 °C. ^1^H-NMR (250 MHz, CDCl_3_): δ 1.14 (12H, app. tt, *J* = 7.0 Hz, *J* = 1.5 Hz, CH_3_), 3.12 (8H, q, *J* = 7.0 Hz, CH_2_), 7.35–7.48 (6H, m, Ar-H), 7.92–8.02 (4H, m, Ar-H). ^13^C-NMR (62.9 MHz, CDCl_3_): δ 7.3, 52.4, 80.0, 128.5 (d, *J* = 14.3 Hz), 130.7 (d, *J* = 12.5 Hz), 131.5, 134.7 (d, *J* = 97.0 Hz). ^31^P-NMR (101.1 MHz, CDCl_3_): δ 17.9. FTIR: 3627 (w), 3055 (w), 2991 (m), 2360 (m), 2141 (m), 1480 (s), 1438 (s), 1395 (m), 1174 (s), 1101 (s), 1034 (s) cm^−1^. HRMS calcd. for C_14_H_10_BSF_3_P [Ph_2_PSCCBF_3_^−^]: 309.0278 Found: 309.0286. 

### 3.7. Synthesis of Tetraethylammonium ((Diphenylphosphoryl)ethynyl)-trifluoroborate **7**

A 30% solution of H_2_O_2_ (0.122 mL) was added to a suspension of potassium [(diphenylphosphino)ethynyl]trifluoroborate **4** (530 mg, 1.67 mmol) in a 1:1 mixture of THF and water (1 mL). The reaction was stirred at room temperature for 16 hours. Then, 2 mL of DCM and a 25% by volume solution of tetraethylammonium hydroxide in water (1.02 mL, 1.80 mmol) were added to the reaction mixture. The biphasic mixture was stirred for 30 min. The organic layer was separated and the aqueous layer washed with DCM (2 × 5 mL). The combined organic fractions were dried over MgSO_4_, and the solvent evaporated to give tetraethylammonium [(diphenylphosphoryl)ethynyl]-trifluoroborate (602 mg, 85%) as a colourless oil. ^1^H-NMR (250 MHz, CDCl_3_): δ 1.20 (12H, app. tt, *J* = 7.0 Hz, *J* = 1.5 Hz, CH_3_), 3.19 (8H, q, *J* = 7.0 Hz, CH_2_), 7.36–7.54 (6H, m, Ar), 7.80–7.92 (4H, m, Ar). ^13^C-NMR (62.8 MHz, CDCl_3_): δ 7.3, 52.4, 128.4 (d, *J* = 13.0 Hz), 128.8, 130.8 (d, *J* = 11.0 Hz), 131.7 (d, *J* = 2.5 Hz), 134.2 (d, *J* = 120.0 Hz). ^31^P-NMR (101.1 MHz, CDCl_3_) δ 7.4. FTIR: 3,619 (w), 3436 (m), 2992 (m), 2144 (m), 1485 (m), 1439 (s), 1396 (m), 1184 (s), 1121 (s), 1035 (s), 975 (s) cm^−1^. HRMS calcd. for C_14_H_10_BOF_3_P [Ph_2_POCCBF_3_^−^]: 293.0514. Found: 293.0514.

### 3.8. Synthesis of 4-((Diphenylphosphoryl)ethynyl)benzonitrile **8a**

Potassium [(diphenylphosphino)ethynyl]trifluoroborate **4** (158 mg, 0.50 mmol, 1 equiv), 4-bromobenzonitrile (91 mg, 0.50 mmol, 1 equiv), PdCl_2_(dppf).CH_2_Cl_2_ (37 mg, 0.045 mmol, 9 mol %), and caesium carbonate (489 mg, 1.50 mmol, 3 equiv) were mixed with dry THF (5.0 mL) and degassed water (0.25 mL) under argon (20:1 THF to water ratio). The solution was heated at reflux overnight. After cooling, 10 mL of water were added, and the resulting solution was extracted with ethyl acetate. The combined organic extracts were washed with brine, dried over MgSO_4_ and the solvent evaporated under reduced pressure. The crude mixture was redissolved in a 1:1 mixture of THF and water, and a 30% solution of H_2_O_2_ (0.06 mL, 0.5 mmol, 1 equiv) was added. After 7 h, the organic layer was extracted, washed with brine and dried over MgSO_4_, and the solvent was evaporated under reduced pressure. The resulting oil was purified by flash chromatography on silica gel (starting with petrol ether, ending with ethyl acetate) to afford 4-[(diphenylphosphoryl)ethynyl]benzonitrile (84 mg, 51%) as a brown solid. M.p. 144–146 °C. ^1^H-NMR (400 MHz, CDCl_3_): δ 7.47–7.51 (4H, m, Ar), 7.54–7.58 (2H, m, Ar), 7.65–7.66 (4H, m, Ar), 7.83–7.89 (4H, m, Ar). ^13^C-NMR (100.6 MHz, CDCl_3_): δ 86.5 (d, *J* = 161.5 Hz), 102.4 (d, *J* = 28.0 Hz), 114.0, 117.8, 124.6 (d, *J* = 4.0 Hz), 128.8 (d, *J* = 13.0 Hz), 131.0 (d, *J* = 11.0 Hz), 131.6, 132.2, 132.6 (d, *J* = 3.0 Hz), 133.0 (d, *J* = 1.5 Hz). ^31^P-NMR (162.0 MHz, CDCl_3_) δ 8.5. FTIR: 3452 (w), 3058 (m), 2229 (m), 2180 (s), 1603 (w), 1499 (m), 1438 (s), 1204 (s), 1122 (s), 852 (s), 725 (s) cm^−1^. HRMS calcd. for C_21_H_15_NOP: 328.0891. Found: 328.0886.

### 3.9. Synthesis of ((4-Nitrophenyl)ethynyl)diphenylphosphine Oxide **8b**

Potassium ((diphenylphosphino)ethynyl)trifluoroborate **4** (158 mg, 0.50 mmol, 1 equiv), 1-bromo-4-nitrobenzene (101 mg, 0.50 mmol, 1 equiv), PdCl_2_(dppf).CH_2_Cl_2_ (37 mg, 0.045 mmol, 9 mol %) and caesium carbonate (489 mg, 1.50 mmol, 3 equiv) were mixed with dry THF (5.0 mL) and degassed water (0.25 mL) under argon (20:1 THF to water ratio). The solution was heated at reflux overnight. After cooling, 10 mL of water were added, and the resulting solution was extracted with ethyl acetate. The combined organic extracts were washed with brine, dried over MgSO_4_ and the solvent evaporated under reduced pressure. The crude mixture was redissolved in a 1:1 mixture of THF and water, and a 30% solution of H_2_O_2_ (0.06 mL, 0.5 mmol, 1 equiv) was added. After 7 h, the organic layer was extracted, washed with brine and dried over MgSO_4_, and the solvent was evaporated under reduced pressure. The resulting oil was purified by flash chromatography on silica gel (starting with petrol ether, ending with ethyl acetate) to afford ((4-nitrophenyl)ethynyl)diphenylphosphine oxide (70 mg, 40%) as a brown solid. M.p. 158–160 °C. ^1^H-NMR (400 MHz, CDCl_3_): δ 7.48–7.53 (4H, m, Ar), 7.55–7.59 (2H, m, Ar), 7.73–7.76 (2H, m, Ar), 7.84–7.90 (4H, m, Ar), 8.20–8.24 (2H, m, Ar). ^13^C-NMR (125.8 MHz, CDCl_3_): δ 87.8 (d, *J* = 161.0 Hz), 102.0 (d, *J* = 27.5 Hz), 123.8, 126.5 (d, *J* = 4.0 Hz), 128.9 (d, *J* = 14.0 Hz), 131.0 (d, *J* = 11.5 Hz), 131.7, 132.7 (d, *J* = 4.0 Hz), 133.5 (d, *J* = 1.5 Hz), 148.5. ^31^P-NMR (162.0 MHz, CDCl_3_) δ 8.5. FTIR: 3661 (w), 3059 (m), 2181 (s), 1594 (m), 1521 (s), 1438 (m), 1345 (s), 1204 (s), 1122 (m), 865 (s), 708 (s) cm^−1^. HRMS calcd. for C_20_H_15_NO_3_P: 348.0790. Found: 348.0797.

## 4. Conclusions

In summary, we report the synthesis and structural characterisation of a novel class of phosphine-substituted alkynylboronates. Moreover, we have shown that this chemistry can be readily extended to the corresponding trifluoroborates, these latter reagents being more stable than the corresponding boronic esters.
